# The impact of the HIRA histone chaperone upon global nucleosome architecture

**DOI:** 10.4161/15384101.2014.967123

**Published:** 2015-01-20

**Authors:** Csenge Gal, Karen M Moore, Konrad Paszkiewicz, Nicholas A Kent, Simon K Whitehall

**Affiliations:** 1 Institute for Cell & Molecular Biosciences; Newcastle University; Newcastle upon Tyne, UK; 2 Biosciences, College of Life & Environmental Sciences; University of Exeter; Exeter, UK; 3 Cardiff School of Biosciences; Cardiff University; Cardiff, UK

**Keywords:** Chromatin, heterochromatin, HIRA, histone chaperone, nucleosome assembly, *S. pombe*

## Abstract

HIRA is an evolutionarily conserved histone chaperone that mediates replication-independent nucleosome assembly and is important for a variety of processes such as cell cycle progression, development, and senescence. Here we have used a chromatin sequencing approach to determine the genome-wide contribution of HIRA to nucleosome organization in *Schizosaccharomyces pombe*. Cells lacking HIRA experience a global reduction in nucleosome occupancy at gene sequences, consistent with the proposed role for HIRA in chromatin reassembly behind elongating RNA polymerase II. In addition, we find that at its target promoters, HIRA commonly maintains the full occupancy of the −1 nucleosome. HIRA does not affect global chromatin structure at replication origins or in rDNA repeats but is required for nucleosome occupancy in silent regions of the genome. Nucleosome organization associated with the heterochromatic (*dg-dh*) repeats located at the centromere is perturbed by loss of HIRA function and furthermore HIRA is required for normal nucleosome occupancy at Tf2 LTR retrotransposons. Overall, our data indicate that HIRA plays an important role in maintaining nucleosome architecture at both euchromatic and heterochromatic loci.

## Introduction

Nucleosome assembly is believed to occur in a step-wise manner whereby the deposition of an (H3-H4)_2_ tetramer is followed by the assembly of 2 flanking H2A-H2B dimers.^1,2^ This process is regulated by a structurally diverse group of proteins termed histone chaperones.^1,2^ Traditionally, these proteins have been classified as either H3-H4 or H2A-H2B chaperones based upon their histone binding specificity, although some chaperones such as FACT are able to bind both H3-H4 and H2A-H2B.^1^ During S-phase nucleosomes are removed ahead of the replication fork and then reassembled onto newly synthesized DNA. However other processes such as transcription, recombination and repair also result in the loss of nucleosomes from DNA which necessitates histone chaperones that mediate replication–independent nucleosome assembly.^3^ Furthermore, it is now established that in addition to their traditional assembly function, histone chaperones can also mediate nucleosome disassembly and histone exchange. Indeed the central role played by histone chaperones in nucleosome dynamics is becoming increasingly recognized.^2^

The HIRA (or HIR) complex is an evolutionarily conserved H3-H4 histone chaperone that is implicated in a range of processes including embryonic development, angiogenesis, cellular senescence and aging.^4^ The human complex is composed of HIRA in association with UBN1 and CABIN1^5-7^ and similarly yeast HIRA proteins (Hir1 and Hir2 in *Saccharomyces cerevisiae* and Hip1 and Slm9 in *Schizosaccharomyces pombe*), are stably associated with orthologs of CABIN1 and UBN1.^8-11^ HIRA co-operates with another H3-H4 chaperone, Asf1 to mediate replication-independent nucleosome assembly.^4^ Consistent with this, in higher eukaryotes HIRA is associated with the histone variant H3.3 which is deposited into chromatin independently of DNA synthesis.^6^

The modulation of chromatin structure by HIRA has been implicated in multiple aspects of transcriptional regulation. In some contexts HIRA is necessary for transcriptional activation. For example the induction of *Vegfr1* in human endothelial cells in response to angiogenic signals is HIRA-dependent.^12^ Similarly in fission yeast, HIRA subunits are recruited to promoters of specific genes in response to environmental stress. Inactivation of HIRA compromises nucleosome eviction and transcriptional induction at these genes.^13^ Conversely, HIRA has also been shown to be required for transcriptional repression. *S. cerevisiae* Hir1 and Hir2 were initially characterized as repressors of histone gene expression,^14^ a role which is conserved in other organisms.^15,16^ Furthermore, HIRA is required for the integrity of silent chromatin in a variety of systems. In fission yeast HIRA/Asf1 spreads across heterochromatic regions via association with the Heterochromatin Protein (HP1) ortholog, Swi6, to maintain a silent state.^10,15,17^ In human fibroblasts HIRA/Asf1a is required for the formation of senescence associated heterochromatin,^18^ and furthermore HIRA interacts with PRC2 and is implicated in the maintenance of the repressive H3K27me mark at developmentally regulated genes in mouse embryonic stem cells.^19^ HIRA also suppresses the expression of retroelements. Mutation in HIRA components alleviates silencing of *S. pombe* Tf2 LTR retrotransposons^10,20^ and human HIRA was revealed as one of a group of chromatin assembly factors that suppresses HIV-1 proviral expression to maintain latency.^21^ Other studies have also suggested a global role for HIRA in transcriptional elongation and the suppression of cryptic promoters. In *S. cerevisiae* *hir* mutations are synthetically lethal when combined with mutations in the yFACT complex which facilitates transcription elongation.^22^ Furthermore inactivation of the HIRA complex results in increased levels of spurious transcripts from cryptic promoters in ORFs.^20,23,24^ The genomes of cells defective in HIRA function also exhibit increased accessibility to DNA damaging agents and nucleases.^20,25^ Taken together the data indicate that the HIRA histone chaperone plays an important role in maintaining the global integrity of chromatin.^20,25^ Given this we have identified the impact of the fission yeast HIRA complex on genome-wide nucleosome architecture. Using a chromatin-sequencing approach^26^ we have mapped changes to nucleosome position and occupancy in cells lacking HIRA function. We find that HIRA is required for normal nucleosome occupancy over ORFs, at some promoters, and also at heterochromatic repeats. As such, HIRA plays an important role in the maintenance of global nucleosomal architecture.

## Results

**Figure 1.**
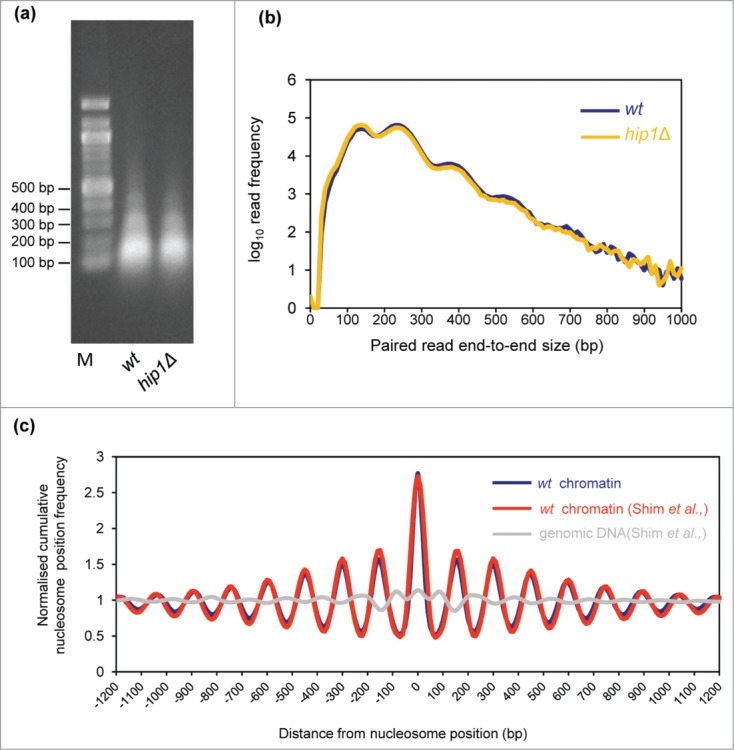
Paired-end mode chromatin-seq of wild type and *hip1*Δ mutant *S. pombe*. (**A**) Ethidium-stained gel separation of DNA pools extracted from MNase digested *S. pombe* chromatin used for chromatin sequencing in this study. Mono-, di- and tri-nucleosomal bands are visible. (**B**) Frequency distribution of paired read end-to-end size values after chromatin-seq of DNA shown in (**A**). (**C**) Nucleosomes in wild type cells (wt chromatin) were defined as the positions of 150 bp size class particle frequency peak summits (frequency value >25). This procedure marked 60, 658 putative nucleosome positions in the *S. pombe* genome. The 150 bp size class particle frequency distribution centered on, and surrounding (+/−1200 bp) each of these positions was then summed and normalized to the average frequency value occurring in the +/−1200 bp window. The wavelength of the peak pattern should be equal to the *S. pombe* nucleosome repeat length. Comparison to a previously published MNase-treated naked DNA (genomic DNA) dataset and a wt chromatin data set ^27^ is shown.

We employed a chromatin sequencing technology^26^ to determine the impact of the HIRA histone chaperone complex on genome-wide nucleosome occupancy and positioning. With this approach, chromatin is treated with micrococcal nuclease (MNase) to generate ladders of MNase-resistant DNA which is then subjected to sequencing. The resulting datasets are then stratified based on paired read end-to-end distance into ranges representing the expected sizes of MNase resistant DNA species in eukaryotic chromatin. Thus read pairs of 150 bp (+/− 20%) derive primarily from mono-nucleosomes, whereas read pairs of 300 bp (+/− 20%) derive from di-nucleosomes. Frequency distributions of the read midpoints can then be mapped to the genome and the peaks in these distributions used to infer the presence of positioned chromatin particles in the cell population.^26^ Therefore chromatin derived from fission yeast cells lacking HIRA function (*hip1*Δ) was digested with MNase to generate a DNA ladder with a highly similar molecular weight distribution to our wild type control sample (**Fig. 1A and B**). Three biological replicate samples were pooled and sequenced which generated data sets for wild type and *hip1*Δ comprising of 56.3 and 49.6 million reads, respectively.

We first compared the average distribution of nucleosomes mapped in our wild-type data-set with that from a previously published study.^27^ A cumulative frequency distribution of nucleosome position at, and surrounding nucleosome positions was plotted to assess how closely the datasets matched. This revealed that the distribution from our wild-type data set is coincident with the previously published wild-type nucleosome data-set and is distinct from a MNase-digested genomic DNA control^27^ (**Fig. 1C**). These control comparisons, suggest that the nucleosome positions we map are accurate and that our wild-type data set agrees well with published work.

### HIRA and the integrity of chromatin associated with ORFs

**Figure 2.**
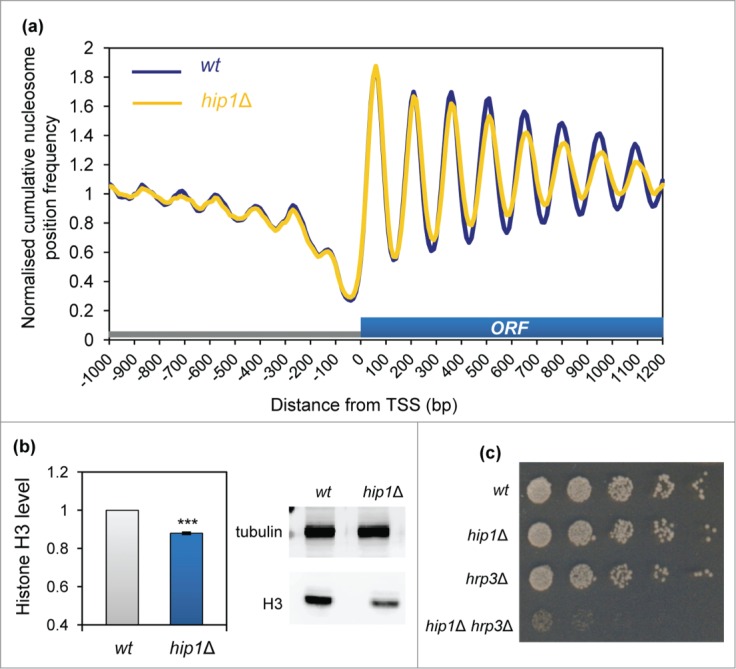
HIRA is required for normal nucleosome occupancy at Pol II transcribed genes. (**A**) Average nucleosome (150 bp size class particle) sequence read frequency profiles for 4013 *S. pombe* genes aligned at the transcription start site (TSS). (**B**) Whole cell extracts were subjected to western blotting with histone H3 (Abcam) and tubulin antibodies. An example of the primary data is shown along with a quantification of histone H3 levels normalized to tubulin (right). Data are the mean of 9 independent repeats and error bars represent ±SEM. *** indicates *P* < 0.001 t-test. (**C**) Strains, NT5 (wt), AW046 (*hrp3*Δ), SW700 (*hip1*Δ), CsG349 (*hip1*Δ *hrp3*Δ) were grown in YE5S medium until they reached an OD_595_ = 0.2–0.3. Cultures were subjected to five-fold serial dilution, spotted onto YE5S agar and incubated for 4 d at 30°C.

As HIRA has been linked to a variety of aspects of transcriptional control,^4^ we examined the impact of deletion of *hip1*^+^ upon the chromatin surrounding the transcription start-sites (TSS) of protein coding genes. Typically chromatin in these regions is organized with a nucleosome depleted region (NDR) followed by a well ordered nucleosome array that extends from the TSS and packages the transcribed region.^28^ In comparison, promoter regions are generally associated with lower nucleosome levels. **Figure 2A** shows a comparison of average nucleosome positions surrounding TSS in wild-type and *hip1*Δ cells. Loss of HIRA did not result in any changes to the NDR or the +1 nucleosome peak indicating that HIRA is not required for the maintenance of chromatin structure around the 5′ end of genes, at least at a global level. Nonetheless, the amplitudes of the nucleosome peaks from +4 onwards were reduced indicating that HIRA does contribute to the maintenance of chromatin associated with ORFs.

The reduction in the average peak height in the *hip1*Δ mutant was suggestive of a decrease in nucleosome occupancy and consistent with this view western blotting revealed a significant reduction in histone protein levels in cells lacking HIRA (**Fig. 2b**). Based upon this finding we predicted that the *hip1*Δ allele would show a strong genetic interaction with mutations in hrp3^+^ which encodes a CHD ATP-dependent remodeler that controls nucleosome spacing.^27,29^ Analysis of a *hip1*Δ *hrp3*Δ double mutant revealed that this strain was extremely slow growing and had severely elongated cell morphology (**Fig. 2C**; **Fig. S1**) Therefore as predicted, loss of correct nucleosome spacing exacerbates the growth defects associated with HIRA inactivation.

**Figure 3.**
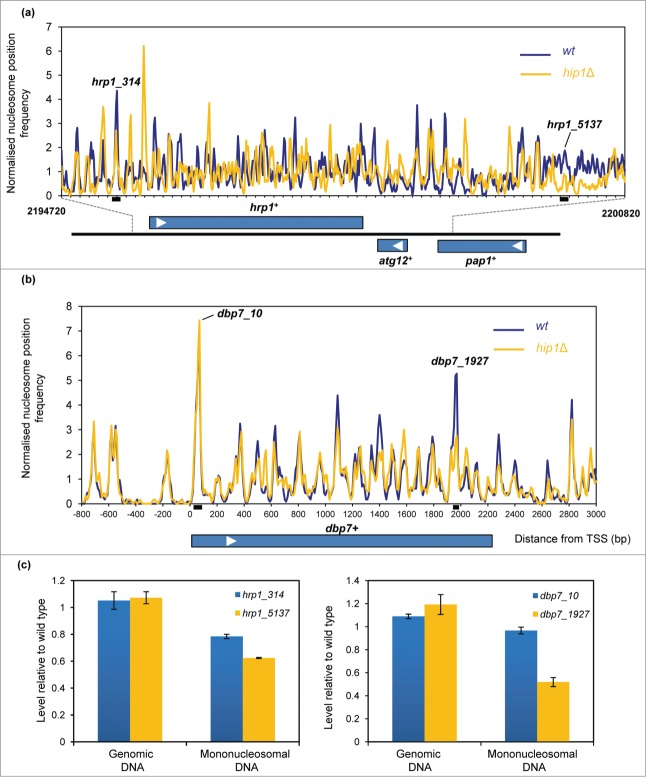
HIRA and nucleosome architecture in gene sequences. (**A**) Nucleosome (150 bp) sequence read frequency profiles of a 6.1 kb region of chromosome 1 (bp 2194720 to 2200820). The positions and orientation of the *hrp1*^+^, *atg12*^+^ and *pap1*^+^ genes are indicated below. (**B**) Nucleosome (150 bp) sequence read frequency profiles of the *dbp7*^+^ gene relative to the TSS. (**C**) The occupancy of specific nucleosomes was estimated by qPCR analysis of mononucleosomal DNA as described in the Materials and Methods. An equivalent amount of genomic DNA was analyzed as a control. The positions of the nucleosome peaks under analysis and the PCR primers are indicated in (**A** and **B**). The level of occupancy in *hip1*Δ relative to wild type is shown. Data is the mean of 2 technical qPCR repeats.

HIRA suppresses aberrant transcription from the bodies of genes^20^ and so the global perturbation to genic chromatin that is observed in the *hip1*Δ strain is consistent with this finding. To further investigate this we analyzed the nucleosome profiles of a group of genes which have been shown to produce cryptic transcripts when HIRA function is absent.^17,20^ At the *hrp1*^+^ locus loss of HIRA function resulted in marked changes to the MNase profile which extended throughout the entire gene and into the neighboring genes (*atg12*^+^ and *pap1*^+^) (**Fig. 3A**; **Fig. S2**). In contrast, the other genes we inspected exhibited relatively modest changes to their nucleosome profiles in the *hip1*Δ background (**Fig. 3B**; **Fig. S3**). An example of this is the *dbp7*^+^ gene, where changes to nucleosome occupancy were mainly observed at the 3′-end of the gene and downstream of the transcription termination site. It therefore appears that relatively small changes to nucleosome architecture may be sufficient to result in increased levels of cryptic transcripts.

In order to confirm some of the differences in the nucleosome profiles that were observed in **Fig. 3A and B**, mononucleosomal DNA was isolated from independent pools of MNase-digested DNA and a quantitative PCR (qPCR) approach^30^ was used to compare the occupancy of some specific nucleosomes in the wild-type and *hip1*Δ samples. Similar to the results from the genome-wide mapping studies, the qPCR analysis also indicated that occupancy of nucleosomes near *hrp1*^+^ (designated hrp1_5137) and the 3′ end of the *dbp7*^+^ gene (dbp7_1927) were reduced in *hip1*Δ whereas the occupancy of nucleosome located at the 5′ (dbp7_10) was similar in wild type and *hip1*Δ.

### Impact of HIRA on chromatin at promoters

**Figure 4.**
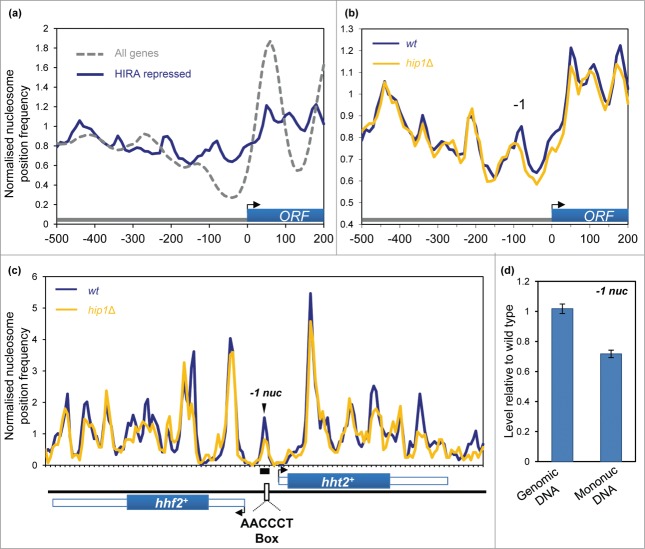
Impact of HIRA on promoter nucleosome profiles. (**A**) Average nucleosome profiles for 4013 *S. pombe* genes aligned at the transcription start site (TSS) compared to the nucleosome sequence read frequency profile of a set of 107 HIRA-repressed protein-coding genes.^20^ (**B**) Average nucleosome profiles of a set of 107 HIRA-repressed genes in wild type and *hip*1Δ cells. **(C)** Comparison of the nucleosome profile at the *hht2*^+^-*hhf2*^+^ locus in wild type and *hip1*Δ cells. The positions of the coding sequences are indicated by solid blue boxes while 5′ and 3′ UTRs are represented by open boxes. Positions of *hht2*^+^-*hhf2*^+^ transcription start-sites, termination sites and the AACCCT box are as described by Takayama and Takahashi.^30^ (**D**) Occupancy of the hht2^+^-hhf2^+^ −1 nucleosome (−1 nuc) was determined by qPCR analysis of mononucleosomal DNA. The position of the PCR primers and peak are indicated in (**C**). The level of occupancy in *hip1*Δ relative to wild type is shown. Data is the mean of 2 technical qPCR repeats.

In addition to the suppression of spurious transcription initiation, HIRA represses expression from numerous bona fide RNA polymerase II (Pol II) promoters. Indeed the expression of approximately 4% of fission yeast genes is increased by loss of HIRA function.^20^ We first compared the chromatin organization of these ‘HIRA-repressed’ genes with the complete set of *S. pombe* coding genes. HIRA-repressed genes were found to have obvious differences in their chromatin organization as average nucleosome peaks associated with the coding sequences of these genes were poorly ordered and the height of the peaks was lower than the global average (**Fig. 4A**). This suggests that the coding sequences of HIRA-repressed genes are associated with a lower than average nucleosome occupancy. Furthermore, the NDR of the HIRA-repressed gene set was both narrower and shallower when compared to the average promoter. These features are known to be characteristic of genes that have a low level of expression,^31^ a finding which is in agreement with our previous microarray analyses which revealed that HIRA target genes overlap significantly with lowly expressed genes.^20^ We next determined the impact of *hip1*^+^ deletion upon chromatin architecture of HIRA-repressed genes. Loss of *hip1*^+^ resulted in a reduction in the height of the −1 nucleosome peak and subtle shift in its position which suggests that HIRA promotes the proper occupancy of the −1 nucleosome, a finding which is consistent with the repressive function of HIRA at these promoters (**Fig. 4B**).

We next examined the histone H3-H4 genes *hht2*^+^-*hhf2*^+^ as their expression outside of S-phase is repressed in a HIRA-dependent manner.^32^ *hht2*^+^-*hhf2*^+^ are divergently transcribed from a short promoter, and analysis of our MNase profiles revealed the presence of a −1 nucleosome peak in the center of this region. Since the majority of fission yeast cells in an asynchronous culture will be in G2, this chromatin configuration is likely to represent a repressed promoter state. Consistent with this hypothesis the promoter nucleosome peak occludes the AACCCT box which is a binding site for the GATA-type factor, Ams2 and the Myb domain protein, Teb1, which activate the transcription of histone genes.^32,33^ Interestingly, both the MNase profiles and qPCR analysis of mononucleosomal DNA indicated that deletion of *hip1*^+^ resulted in a marked reduction of this peak, suggesting that HIRA promotes the occupancy of this promoter nucleosome to suppress the inappropriate expression of histone genes (**Fig. 4C and D**).

### HIRA is not required for chromatin architecture at Pol I- and Pol III-transcribed genes

**Figure 5.**
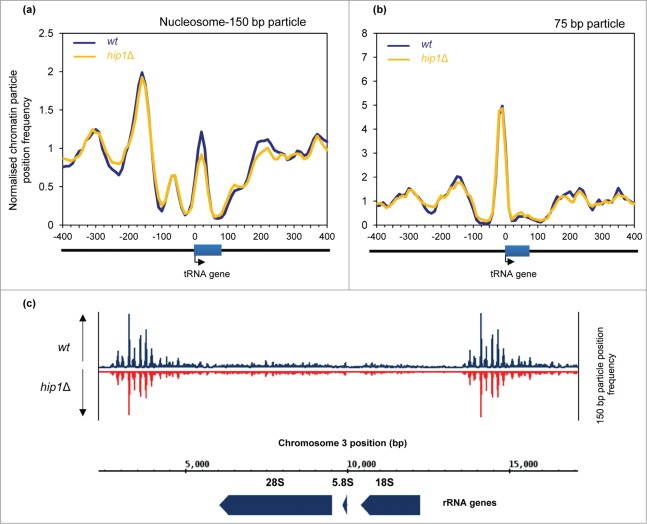
Nucleosome architecture at Pol III and Pol (**I**)genes. (**A**) Average nucleosome (150 bp size class particle) sequence read frequency profiles for 171 *S. pombe* tRNA genes aligned at the transcription start site (TSS). (**B**) Average 75 bp size class particle sequence read frequency profiles for 171 *S. pombe* tRNA genes aligned at the transcription start site (TSS). **(C)** Nucleosome (150 bp size class particle) read profile over an rDNA repeat. The positions of the 28S, 5.8S and 18S rRNA genes are indicated.

As HIRA has a global impact upon the chromatin associated with Pol II-transcribed genes we examined whether it was also required for chromatin organization at Pol I and Pol III genes. We analyzed the chromatin configuration surrounding Pol III-transcribed tRNA genes. In *S. cerevisiae* tRNA genes are typically nucleosome free and flanked by nucleosomes positioned upstream (US) and downstream (DS).^34^ In comparison, it has been suggested that many tRNA genes in *S. pombe* (like those in resting human CD4+ T cells) are associated with nucleosomes.^34^ Plots of the average mono-nucleosome (150 bp) profile of 171 *S. pombe* tRNA genes aligned by TSS are consistent with this earlier report as we detected a peak centered at +20 relative to the TSS (**Fig. 5A**). At this global level we were able to detect an upstream (US) nucleosome peak positioned at −160 bp but we found little evidence of a downstream nucleosome array. Loss of HIRA did not impact upon the US nucleosome although we did note some reduction in the height of the peak located at +20 bp. tRNA genes have internal promoter elements that are binding sites for TFIIIC which in turn directs the assembly of TFIIIB upstream of the transcription start-site. TFIIIB acts as the initiation factor by bringing Pol III to DNA.^35^ In order to see if HIRA has any global impact upon Pol III transcription factor binding we examined the profile of 75 bp particles as it has been demonstrated that these particles result from the protection of DNA by transcription factors rather than by nucleosomes.^26^ Comparison of average 75 bp profiles revealed the presence of a prominent peak immediately upstream of the TSS which given its position is likely to result from TFIIIB binding (**Fig. 5B**). Loss of HIRA function did not impact upon this peak suggesting that it does not globally affect TFIIIB occupancy at tRNA genes.

Recently, the HIR complex has been implicated in the repression of rDNA transcription in *S. cerevisiae*.^36^ We therefore analyzed the MNase profiles of *S. pombe* rDNA repeat sequences. This suggested that Pol I-transcribed genes are nucleosome free while the intergenic regions are associated with well-positioned nucleosomes. However, loss of HIRA did not have a marked affect upon the MNase profiles of these regions (**Fig. 5C**) and so we find no evidence to suggest that HIRA is required for the maintenance of nucleosome architecture at Pol I genes in fission yeast. We also examined nucleosome profiles surrounding replication origins which were aligned as described previously.^37^ In agreement with previous studies,^31,37^ a wide nucleosome depleted region (NDR) was detectable over the origin center. This feature was also readily detectable in *hip1*Δ cells and indeed the average MNase profile of wild-type and *hip1*Δ cells surrounding origins was strikingly similar (**Fig. S4**) suggesting that HIRA does not contribute to the global organization of chromatin at replication origins.

### Impact of HIRA upon nucleosome organization in silent chromatin

**Figure 6.**
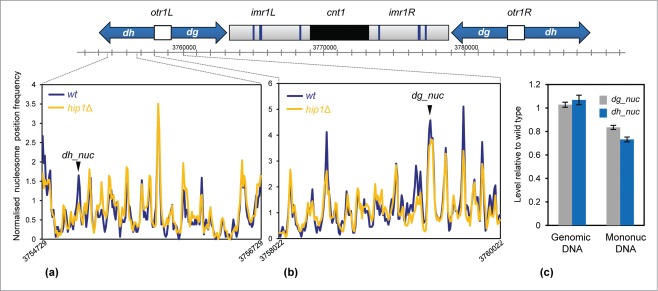
Loss of HIRA perturbs nucleosome architecture at centromeric repeats. (**A and B**) A schematic diagram of centromere 1 is shown along with the average nucleosome (150 bp) sequence read frequency profiles of the indicated regions of the *dg* and *dh* repeats. (**C**) The occupancy of specific *dh* and *dg* repeat nucleosomes was estimated by qPCR analysis of mononucleosomal DNA as described in the Materials and Methods. An equivalent amount of genomic DNA was analyzed as a control. The positions of the nucleosome peaks under analysis are indicated in (**A and B**). The level of occupancy in *hip1*Δ relative to wild type is shown. Data is the mean of 2 technical qPCR repeats.

Loss of any one of the subunits of the HIRA complex alleviates heterochromatic silencing at the cryptic mating (*mat*) type locus and also at pericentromeric repeats.^8,10^ These heterochromatin domains are enriched for methylation of histone H3 lysine 9 (H3K9me) which directs the assembly of chromodomain proteins such as the HP1 ortholog, Swi6.^38^ Hip1 interacts with Swi6 and also the histone chaperone Asf1, which is required for nucleosome occupancy in heterochromatin.^17^ We therefore examined the impact of *hip1*^+^ deletion upon the nucleosome profile at pericentromeric *dg-dh* repeats. This revealed that loss of HIRA resulted in changes to specific peaks rather than a uniform reduction in occupancy across the entire repeat region (**Fig. 6A and B**). Consistent with this, qPCR analysis also indicated that occupancy of specific dg and dh nucleosomes (designated dh_nuc and dg_nuc) were reduced in the absence of HIRA (**Fig. 6C**). This suggests that HIRA is required to maintain the proper occupancy of a subset of nucleosomes within heterochromatic domains and that this is required for transcriptional silencing in this region.

**Figure 7.**
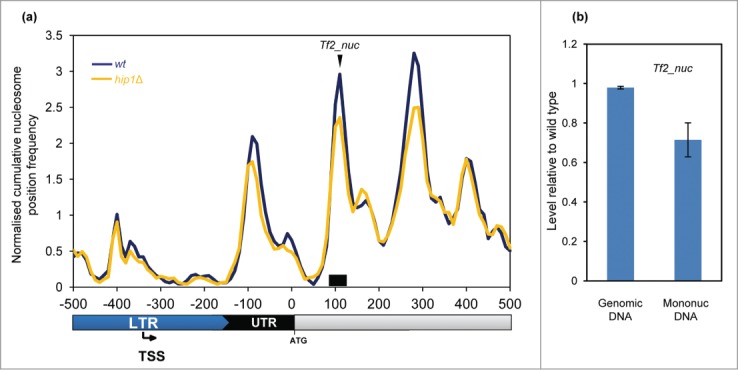
Nucleosome structure of Tf2 LTR retrotransposons. (**A**) Average nucleosome (150 bp) sequence read frequency profile for the 5′ region of Tf2 elements aligned relative to the translation start site (ATG). (**B**) The occupancy of a specific Tf2 nucleosome was estimated by qPCR analysis of mononucleosomal DNA as described in the Materials and Methods. An equivalent amount of genomic DNA was analyzed as a control. The position of the nucleosome peak and the PCR primers are indicated in (**A**). The level of occupancy in *hip1*Δ relative to wild type is shown. Data is the mean of 2 technical qPCR repeats.

HIRA is also required for silencing the expression of all 13 intact Tf2 LTR retrotransposons.^20^ The mechanism of silencing of these elements is distinct from heterochromatin as although it requires HIRA, it is independent of H3K9me.^10,39^ Plots of average nucleosome profiles of these LTR retrotransposons showed that they have a nucleosome architecture which is distinct from typical RNA Pol II-transcribed genes. At Tf2 promoters (5′ LTRs), a peak overlapped the TSS and the NDR was located downstream (rather than upstream) of the TSS (**Fig. 7A**). Interestingly, deletion of *hip1*^+^ had very little affect on the nucleosome peak adjacent to the TSS however we noted that *hip1*Δ cells had reduced +1, +2, and +3 nucleosome peaks (relative to the NDR). qPCR analysis also indicated that deletion of *hip1*^+^ resulted in a reduction in the occupancy of the +2 nucleosome (**Fig. 7B**). These findings suggest that the nucleosomes downstream of the TSS may play a role in repression of Tf2 retrotransposons.

## Discussion

Here we have determined the global impact of HIRA upon nucleosome architecture and in agreement with previous evidence, find that this histone chaperone plays roles in the maintenance of both euchromatic and heterochromatic regions of the genome. We demonstrate that cells lacking HIRA (*hip1*Δ) experience a global reduction in nucleosome occupancy. This is consistent with previous studies which revealed that the genomes of fission yeast HIRA mutants are more accessible to DNA damaging agents.^20^ Similarly in mammalian cells, HIRA depletion results in increased sensitivity of the genome to nucleases.^25^

The co-ordinated replacement of nucleosomes that are displaced by elongating Pol II is necessary for maintaining the integrity of chromatin structures associated with gene sequences.^40^ The finding that cryptic intragenic transcripts increase in the absence of HIRA^20^ is consistent with its proposed role in chromatin reassembly in the wake of Pol II. Our data adds further support to this hypothesis, as average MNase profiles revealed that *hip1*Δ cells have a global reduction in nucleosome occupancy which was most pronounced toward the 3′ end of gene sequences. Reduced levels of specific nucleosome peaks were also detectable at individual genes where loss of HIRA results in increased cryptic transcription. However there are also some regions of the genome which are more severely perturbed in the absence of HIRA. Indeed marked changes to both the occupancy and positioning of nucleosome peaks were evident surrounding the *hrp1*^+^ locus. Why specific regions of the genome show a greater dependency upon HIRA than others is currently not clear.

Comparison of average nucleosome plots aligned by TSS in wild-type and *hip1*Δ cells did not reveal a global impact of HIRA upon chromatin at promoters. However when we analyzed the profiles of a set of HIRA repressed genes^20^ we found that *hip1*^+^ is required for the normal occupancy and positioning of the −1 nucleosome. This set of genes has a chromatin structure that is characteristic of lowly expressed genes^31^ which is consistent with transcript profiling of HIRA mutant cells.^20^ Taken together this suggests that HIRA commonly functions to maintain a closed/repressive chromatin structure at these genes. This was also evident at the *hht2*^+^-*hhf2*^+^ promoter where HIRA is required for full occupancy of the −1 nucleosome peak. *S. pombe* has 3 H3-H4 gene pairs however HIRA-mediated repression of histone gene expression is believed to operate predominantly through this gene pair.^32^ Interestingly, the −1 nucleosome peak occludes the proposed binding site for the Ams2 and Teb1 activators suggesting that remodelling of this nucleosome may be required during transcription activation. Reduced occupancy of this nucleosome in the absence of HIRA would be expected to facilitate binding of activating transcriptions factors. While the expression of *ams2*^+^ is limited to G1/S,^41^ the expression of *teb1*^+^ is constitutive.^42^ Therefore the absence of HIRA may allow increased Teb1 binding and thus expression of *hht2*^+^-*hhf2*^+^ outside of S phase. Our results suggest that HIRA is required for a ‘closed’ chromatin configuration at some promoters and similarly in *S. cerevisiae* Hir1 has been shown to be necessary for chromatin re-assembly at the *PHO5* promoter during the switch from active transcription to repression.^43^ While these results indicate that HIRA plays roles in promoting nucleosome occupancy, in other contexts it is also involved in mediating nucleosome eviction. HIRA subunits are recruited to specific stress-responsive promoters to facilitate nucleosome removal and gene induction.^13^ Furthermore, the MNase profiles suggested that the occupancy of some nucleosomes is increased in the absence of HIRA. Therefore, in common with other histone chaperones, HIRA seems capable of mediating both nucleosome assembly and disassembly.^2^

Chromodomain HP1 proteins such as Swi6 are hallmarks of heterochromatin. It has been proposed that these factors provide a platform for the assembly of other chromatin modifying proteins (including HDACs, ATP-dependent remodelers and histone chaperones) which enforce silencing of the underlying repeat sequences.^38^ HIRA is one of these effectors as its correct localization at heterochromatic repeats is dependent upon Swi6.^17^ Previous studies have shown that changes to nucleosome positioning perturb heterochromatin function.^44^ Here we present data which indicates that the changes in nucleosome occupancy associated with loss of HIRA negatively impact upon heterochromatin silencing. Our MNase profiles indicate that loss of HIRA results in changes to specific nucleosomes rather than a uniform reduction across pericentromeric *dg-dh* repeats. Nonetheless, that cells lacking HIRA have increased levels of centromeric ncRNAs and defective trans-gene silencing,^10,15,17^ implies that these changes are sufficient to impair heterochromatin function and allow increased access of Pol II to repeat sequences. We note that a similar situation has been reported for Hrp3 because loss of this CHD remodeler results in dysfunctional heterochromatin without producing dramatic changes upon nucleosome architecture.^27^

HIRA has been linked to the regulation of both LTR retrotransposons and retroviruses.^20,45,46^ That loss of HIRA function leads to a dramatic increase in expression of Tf2 LTR retrotransposons prompted comparison of MNase profiles of these elements. Tf2 5′-LTR regions are associated with a single nucleosome which overlaps the TSS. HIRA does not have an effect upon this nucleosome but is required for the full occupancy of the nucleosomes downstream of the transcription start-site. This suggests that chromatin structure in this region is important for maintaining silencing of Tf2 retrotransposons. Interestingly, analysis of HIV-1 expression has demonstrated that a nucleosome downstream of the TSS is important for mediating Pol II pausing and suppressing basal expression.^47^ While the integrity of this region is dependent upon the FACT histone chaperone, other analyses revealed that HIRA is also required for the suppression of HIV-1 proviral expression and the maintenance of latency.^45^ Given the parallels between Tf2 and HIV-1 it will be interesting to determine whether Pol II pausing is required for silencing of Tf2 elements.

## Materials and Methods

***S. pombe* strains**

Routine culture and genetic manipulation was performed as previously described.^48^ The strains used in this study were 972 (*h^−^*), SW577 (*h^−^ hip1::ura4^+^*), NT5 (*h^−^ ade6^−^ ura4-D18 leu1–32*), AW046 (*h^+^ hrp3::kanMX ade6^−^ leu1–32 ura4-D18*), SW700 (*h^−^ hip1::ura4^+^ ade6^−^ ura4-D18 leu1–32*), CsG349 (*h^−^ hip1::ura4^+^ hrp3::kanMX ade6^−^ ura4-D18 leu1–32*).

### Histone levels

Approximately ∼4 × 10^7^ cells were harvested following addition of trichloroacetic acid (TCA) to a final concentration of 10%. Cells were resuspended in 200 μl 10% TCA and then disrupted using a beadbeater with 0.75 ml of glass beads using 2 pulses of 15 sec with 1 min on ice in between. A 500 μl aliquot of 10% TCA was added, the lysate was recovered from the beads which was then clarified by spinning at 13 000 rpm in a microcentrifuge. The resulting pellet was washed 3 times in acetone, dried and resuspended in 30 μl 100 mM Tris-HCl (pH 8.0), 1% w/v SDS, and 1 mM EDTA. Samples were analyzed on SDS-PAGE gels and subjected to protein gel blotting using anti-histone H3 (Abcam ab1791) and anti-tubulin (TAT-1) antibodies.

### MNase digestion of chromatin

Cells (100 ml) were grown to OD_595_ = 0.75–8.0 in YE5S at 30°C, crosslinked for 20 min at 30°C using 1% formaldehyde and quenched by the addition of glycine to 125 mM. Cells were washed once in CES buffer (50 mM citric acid/50 mM Na_2_HPO_4_ [pH 5.6], 40 mM EDTA [pH 8.0], 1.2 M sorbitol and 10 mM β-mercaptoethanol) and resuspended in 500 μl of CES buffer with 0.5mg Zymolase 100-T. Cells were spheroplasted at 30°C for up to 1 h and then washed twice with ice cold 1.2 M sorbitol. Spheroplasts were then resuspended in 800 μl NP-S buffer (1.2 M sorbitol, 10 mM CaCl_2_, 100 mM NaCl, 1 mM EDTA pH 8.0, 14 mM β-mercaptoethanol, 50 mM Tris [pH 8.0], 0.075% NP-40, 5 mM spermidine, 0.1 mM PMSF, 1% Sigma Protease inhibitors cocktail [Sigma P8215]). Spheroplasts were then divided into 4 200 μl aliquots and each aliquot was mixed with 300 μl of NP-S buffer. Three aliquots were digested with between 75–187.5 units of MNase (USB) for 10 min at 37°C. The fourth was retained as an undigested control. MNase digestion was terminated by adding EDTA [pH 8.0] to a final concentration of 50 mM and SDS to 0.2%. Reactions were incubated at 65°C overnight with 0.2 mg/ml proteinase K and 10 μg RNAse. DNA was purified by extracting twice with phenol:chloroform followed by ethanol precipitation (0.1 volumes of 3 M sodium acetate followed by 2 volumes of ethanol). Pellets were washed in 70% ethanol and resuspended in water containing 10 μg/ml RNase and incubated at 37°C for 30 min. Triplicate digests were pooled and treated with 100 U unmodified T4 polynucleotide kinase (NEB) for 30 min at 37°C to remove 3′-phosphate groups left by MNase. DNA was extracted once more with phenol:chloroform, re-precipitated with sodium acetate and ethanol, washed with 70% ethanol, dried and re-suspended in TE (pH 7.5).

### Chromatin-seq

DNA fragments were end repaired, 3′-adenylated and ligated to indexed adapters without size selection using Nextflex reagents (Newmarket Scientific, UK). Libraries were amplified with 8 cycles PCR using Kapa HiFi PCR master mix (Anachem), primers removed with GeneRead size selection protocol (QIAgen) before quantification by Bioanalyser DNA 7500 assay. Libraries were pooled, denatured, diluted to 6 nM before clustering in a single lane of a high output Illumina flowcell. Sequencing (100 nt) was undertaken on a HiSeq 2500 using TruSeq SBS v3 reagents (Illumina).

### Bioinformatics

Paired reads were aligned to the ASM294v1.17 reference genome using Bowtie 0.12.7 ^49^ with command line flags: -n 0 –trim3 75 –maxins 5000 –fr -k 1 –sam. Aligned read pairs were sorted according to chromosome and then into a range of size classes based on the SAM format ISIZE value (difference between 5′ end of the mate read and the 5′ end of the first mapped read) plus or minus 20%. Mono-nucleosome-sized reads are, therefore, represented as 150 bp ± 30 bp. To define the genomic position of MNase-resistant chromatin entities we mapped the mid-point position of the read pairs in a particular size class. Frequency distributions of the mid-point positions were then calculated using 10 bp bins. Frequency distributions were lightly smoothed by taking a 3-bin moving average. All frequency distributions were output in the zero-referenced, chromosome base, 3-column .sgr format (chromosome number, feature/bin position, mid-point frequency value) for rendering with the Integrated Genome Browser^50^ and for further processing. Average cumulative chromatin particle position frequency distributions at, and surrounding, genomic features were calculated using the script SiteWriterCFD as described previously,^26,51^ with values for each bin normalized to the average cumulative frequency value obtained for all bins within the feature window. To provide the comparison (**Fig. 1c**) of our data with the smoothed nucleosome position map of Shim and co-workers^27^ the positions of 33874 unambiguous peak summit bins were marked in our wild type KDE mono-nucleosome data set (using script PeakMarkerEpKDE) and compared with GSM994397_WT.wig replicate and GSM994402_genomicDNA.wig data (converted to 10bp binned .sgr format). Protein-coding gene transcription start sites (TSS) positions were as described by Lantermann et al.^31^ Replication origin positions were as described by Givens et al.^37^

### qPCR analysis of mononucleosomal DNA

MNase digests of wild type and *hip1*Δ cells were performed as described above. For each strain 3 biological replicate samples (independent from those used for sequencing) were pooled and analyzed on 1% TAE agarose gels. Gel slices containing mononucleosomal DNA were excised, frozen at -80°C and spun through 0.45 μM Spin-X columns (Costar). Samples were phenol extracted and ethanol precipitated and resuspended in TE (pH7.5). dsDNA concentration was measured using a Qubit fluorometer (Life Technologies). 20 ng of mononucleosomal DNA and was used in qPCR reactions using the PrimerDesign Mastermix kit. Reactions using the equivalent amount of genomic DNA were included as a control. The primers used for this analysis are listed in **Table S1**.
