# Supplementary material for: The impact of the HIRA histone chaperone upon global nucleosome architecture
Source: Cell Cycle. 2015 Jan 20;14(1):123–34. doi: 10.4161/15384101.2014.967123 (PMC4614360; doi:10.4161/15384101.2014.967123)
Supplement: 967123_Supplementary_Materials.zip [file kccy-14-01-967123-s001.zip › 967123_Supplementary Materials/967123_Supplementary Information.pdf]

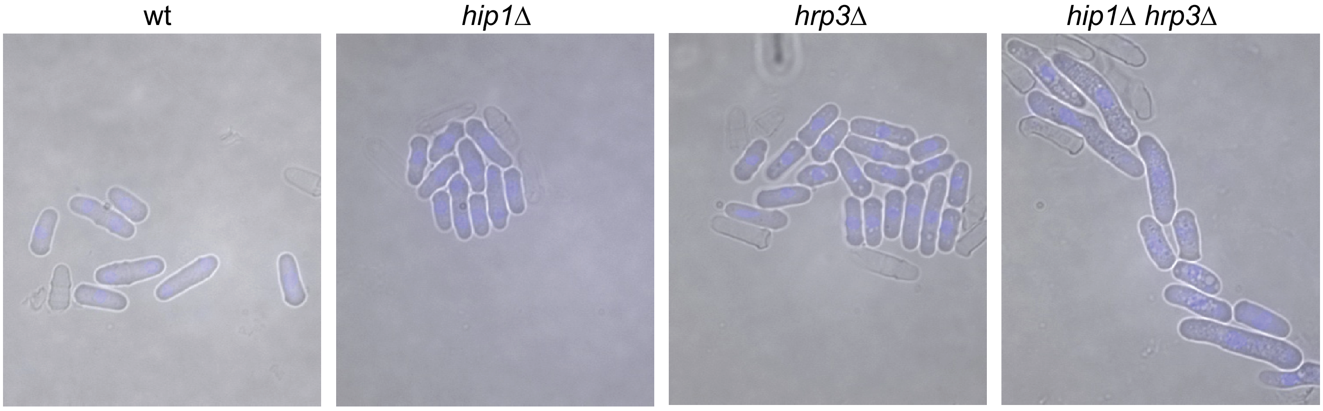

Supplementary Figure 1. Comparison of the morphology of wild type *hrp3*Δ, *hip1*Δ and *hip*Δ *hrp3*Δ strains. Mean cell lengths ± S.D. (µm) were; wild type 10.1 ± 1.8, *hrp3*Δ 10.0 ± 2.2, *hip1*Δ 10.9 ± 2.9 and *hip*Δ *hrp3*Δ 18.02 ± 6.2.

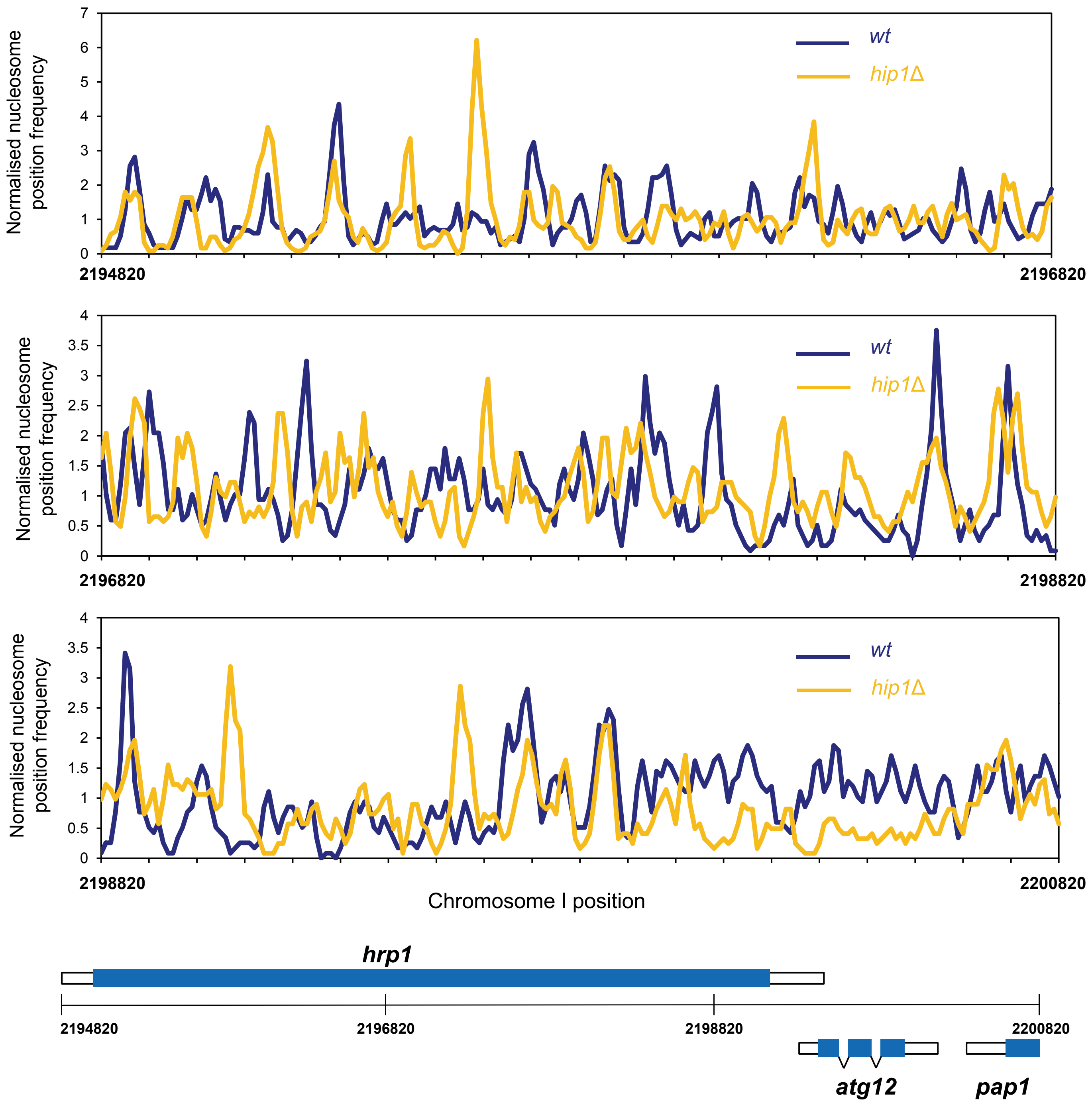

Supplementary Figure 2. Impact of HIRA inactivation upon nucleosome organisation surrounding *hrp1*⁺. Nucleosome (150 bp) sequence read frequency profiles of the indicated regions of chromosome 1. The relative positions and orientation of the *hrp1*⁺, *atg12*⁺ and *pap1*⁺ genes are indicated below. Coding sequences are indicated by solid blue boxes while 5′ and 3′ UTRs are represented by open boxes.

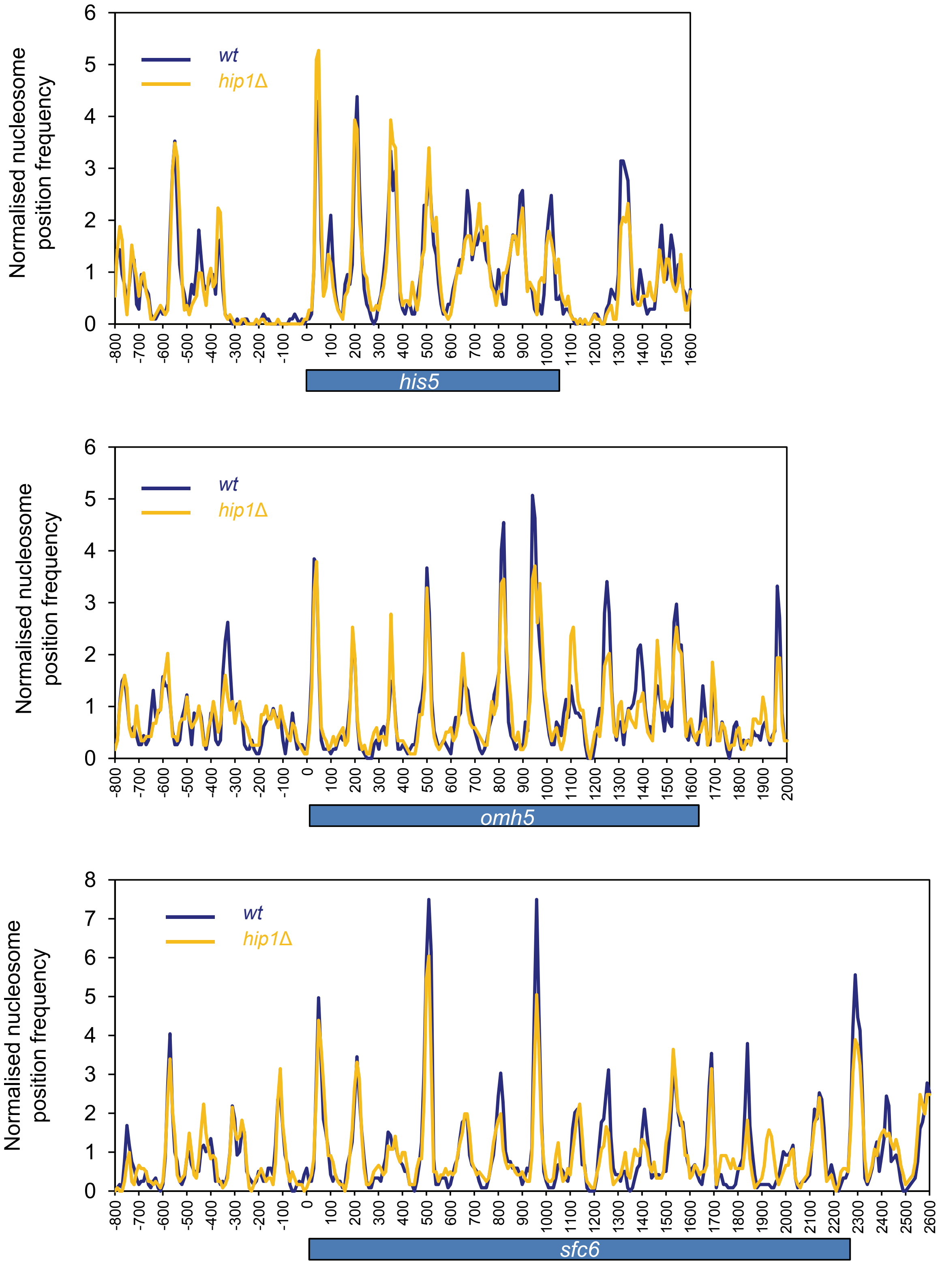

Supplementary Figure 3. Effect of HIRA on nucleosome architecture in gene sequences. Nucleosome (150 bp) sequence read frequency profiles at the indicated gene relative to the TSS.

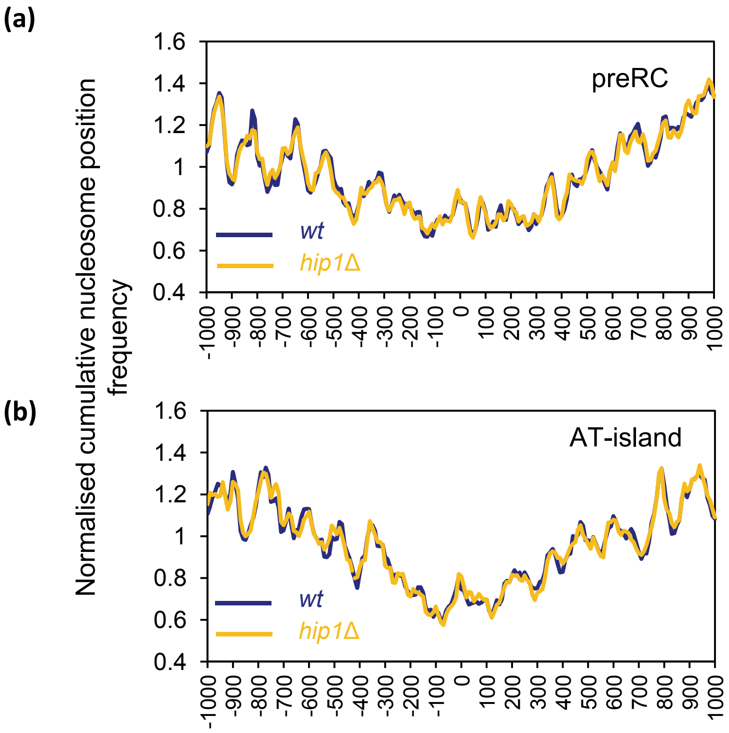

Supplementary Figure 4. Loss of Hip1 does not perturb chromatin associated with replication origins. Average nucleosome sequence read frequency profile surrounding 217 replication origins. **(a)** Origin centre is based upon the site of pre-RC binding and **(b)** AT-island position as described by Givens *et al.*[37]

Supplementary Table 1. Mononucleosomal DNA qPCR primers

**Name**	**Sequence (3′ → 5′)**
dbp7_10 for	ACC ATT GCT TCT CAA TTT TG
dbp7_10 rev	CGA CTA GAC TTC AAG GCT TC
dbp7_1927 for	ATG CGT GAC CTT CAT TTG GG
dbp7_1927 rev	GCG CTT CTC GCA AAG CGA AG
hrp1_5137 for	CTG AGT TAA AAT ACA CAT CTG
hrp1_5137 rev	TAA TAT TCG TCG ACC AAA GG
hrp1_314 for	AAT CAT GAA AAT TCT TTC GC
hrp1_314 rev	ATC ATC AAA GGC AGA AGA CG
hht2_-1 for	GTA GCG GGG AAG CCG AAA TC
hht2_-1 rev	CAA TCA CAA CCC TAA CCC TG
dg_nuc for	AAT TCG GGT CAT ACT TCG TG
dg _nuc rev	CAA TCA TAC TCG AAA AAA AAG AAA TC
dh_nuc for	GTT AAA AGT GGC AGA AAG TG
dh_nuc rev	ATA TGC GTT GGG TTA TCT CA
Tf2_nuc1 for	GAA CAT TTA ATA AAC CTT TTT GC
Tf2_nuc1 rev	ATC GAA TTT CCC TAT CTC TG
